# Is inflammatory potential of the diet related to oral and periodontal health?

**DOI:** 10.1002/fsn3.3641

**Published:** 2023-08-28

**Authors:** Ziya Erokay Metin, M. Merve Tengilimoglu‐Metin, Nuh Oğuz, Mevlüde Kizil

**Affiliations:** ^1^ Department of Nutrition and Dietetics, Gulhane Faculty of Health Sciences University of Health Sciences Ankara Turkey; ^2^ Department of Nutrition and Dietetics, Faculty of Health Sciences Hacettepe University Ankara Turkey; ^3^ Presidential Health Center of Republic of Turkey Ankara Turkey

**Keywords:** diet, dietary inflammatory index, gingival index, inflammation, periodontal diseases, periodontal health

## Abstract

Inflammation is among the risks of periodontal diseases. The relationship between the inflammatory load of the diet and inflammation has been shown in previous studies, but the relationship between periodontal diseases and the inflammatory load of the diet is not clear. In this study, it was aimed to examine the relationship between dietary inflammatory index (DII) and periodontal health. Board approved the protocol. Information about the study was given to the patients who met the criteria of the study and agreed to participate in the study. Oral health status was evaluated by measuring the Decayed, Missing, and Filled Teeth (DMFT). Periodontal health status was determined using the plaque index (PI) and gingival index (GI). Twenty‐four‐hour dietary record was taken for 1 day to calculate the inflammatory load gained from daily diet, and the DII score was used. The study group consisted of 138 participants. DMFT scores were found with median 4.0, and no statistically significant difference was observed between DII quartiles. 65.8% of the participants had absence or small amount of plaque accumulation, while 39.2% had moderate or dense amount of plaque accumulation according to the PI classification. While DII did not differ by PI classification, DII quartile between second and third found a difference according to the GI classification. The new definition of periodontal health has been proposed as the absence of clinically detectable inflammation. It seems that pro‐inflammatory properties of the diet and periodontal health are related. But future randomized controlled trials are needed.

## Graphical abstract

Inflammation is among the risks of periodontal diseases. Oral health status was evaluated by measuring the Decayed, Missing, and Filled Teeth. It seems that pro‐inflammatory properties of the diet and periodontal health are related.

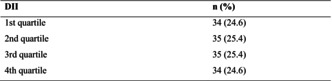

## 1 INTRODUCTION

Nutrition has a major modifiable role in periodontal diseases. Cariogenic dietary habits and poor oral hygiene are key risk factors in dental caries (Sheiham & James, 2015). Dental caries is one of the most common infectious diseases. The caries process begins with the supply of dietary simple sugars (mono and disaccharides) to the bacteria in the dental plaque. Fermentable carbohydrates are called cariogenic foods which serve as the substrate for bacteria, and these carbohydrates are metabolized by bacteria, resulting in the emergence of organic acids in high concentrations, and lead to demineralization of tooth enamel (Mahan & Raymond, 2016; Stanski & Palmer, 2015). If demineralization exceeds remineralization over time, cavitation of the enamel surface will occur, leading to decay (Kleinman & Greer, 2014). The type, form, and frequency of food and drink consumed have a direct effect on oral pH and microbial activity, which can increase tooth decay (Mahan & Raymond, 2016). Studies have shown that the risk of dental caries is low if the per capita simple sugar intake is less than 15 kg per year (Moynihan, 2005). In addition, a diet rich in refined carbohydrates and saturated fat can initiate inflammatory processes through the production of reactive oxygen species. Restricting refined carbohydrates and saturated fats can reduce the activation of pro‐inflammatory pathways (Gondivkar et al., 2019).

Plaque formation is considered to be the main factor in the development of periodontal diseases. As a result of the increase in plaque volume, a suitable environment is created for the development of periodontopathogens. The increase in plaque volume is associated with high sucrose intake, and studies show that high sucrose intake is associated with an increased risk of gingivitis (Gondivkar et al., 2019). Another mechanism for the effect of excessive sugar consumption on periodontal diseases is associated with chronic inflammation. Sugary foods and beverages contribute to high glycemic load, which leads to inflammation, insulin resistance, and impaired beta‐cell function. It has been suggested that these inflammatory changes resulting from the consumption of sugary foods and beverages may lead to an increase in the prevalence of periodontal diseases (Song et al., 2016).

Studies indicate that oxidative stress is an important part of the pathogenesis of periodontal diseases (Muniz et al., 2015). The progression of periodontal diseases seems to depend on the individual's immune response and oxidative sensitivity. Cytokines, which are responsible for the initiation and maintenance of the inflammatory and immune response, transmit all kinds of local and systemic inflammatory responses in the host (Nibali et al., 2012). They play an important role in many chronic inflammatory and systemic diseases, including periodontal diseases, rheumatoid arthritis, and diabetes (Okada & Murakami, 1998). Adipose tissue‐derived cytokines and hormones are thought to have a key role in the formation of periodontal diseases (Saito & Shimazaki, 2007). Among these secreted cytokines, especially tumor necrosis factor‐alpha (TNF‐α), interleukin (IL)‐1 (β and α), and IL‐6 are important for the development of periodontal disease (Okada & Murakami, 1998). Proinflammatory cytokines are known to play a role in the pathogenesis of periodontal disease; IL‐1, IL‐6, IL‐8, IL‐11, IL‐12, IL‐15, IL17, IL‐121, IL‐22, IL‐23, IL‐32, TNF‐α, and prostaglandin E2, anti‐inflammatory cytokines are IL‐1Ra, IL‐4, IL‐10, IL‐12, IL‐13, IL‐18, IL‐27, IL‐33, interferon (IFN)‐α, IFN‐β, and transforming growth factor‐β (Dinarello, 2000; Takashiba et al., 2003). Based on the relationship between inflammatory markers reported in the literature, the dietary inflammatory index (DII) was developed to measure the potential inflammatory effect of a person's diet, and a high DII score indicated a pro‐inflammatory diet. It is well documented that this index correlates with serum C‐reactive protein (CRP) level (Shivappa et al., 2014). In one study, it was shown that there is a relationship between dental hygiene, preventive dental care, dental health, and systemic inflammatory markers (Frisbee et al., 2010). Most of the studies have focused on a protective role of single nutrients to periodontal health or the deleterious effect of cariogenic dietary patterns. However, the pro‐ or anti‐inflammatory effects of the whole diet on the enhance of caries and periodontal disease remain elusive. Therefore, this study was planned to evaluate the relationship between the dental health and the inflammatory index of the diet.

## 2 MATERIALS AND METHODS

### 2.1 Participants

Exclusion criteria included using drugs (antidepressants, metformin, etc.), having conditions affecting appetite, chronic or systemic diseases, psychiatric diseases, and/or receiving eating behavior therapy, pregnancy, or lactation. Non‐Interventional Clinical Researches Ethics Board approved the protocol. Detailed information about the study was given to the participants who met the criteria of the study and agreed to participate in the study.

### 2.2 Oral and dental examination

The determination of oral and dental health was carried out by a dentist. Oral health status was evaluated by measuring the Decayed, Missing, and Filled Teeth (DMFT). Periodontal health status was determined using the plaque index (PI) and gingival index (GI).

### 2.3 Decayed, Missing, and Filled Teeth

DMFT index was determined by using the number of DMFT of the individuals. The DMFT index shows the number of caries per person in a permanent set of teeth.

### 2.4 Plaque index

It evaluates the bacterial plaque and plaque thickness in contact with the marginal gingiva. This index is particularly suitable for examining the effect of bacterial plaque on gingivitis. Measurements are made by rubbing and pulling the periodontal probe on the tooth surface in the gingival pocket. The Silness and Löe PI was used to evaluate the amount of plaque on the teeth. Plaque evaluation on the surface (mesial, distal, labial, lingual) in four regions for each tooth was made by periodontal probe and visual inspection, and each surface was given a value between 0 and 3. The personal PI score is calculated by dividing the total score by the number of tooth surfaces examined; the population PI can be calculated by dividing the total PI by the total number of people. Calculated PI value was classified as absence of plaque, small, moderate, or dense amount of plaque accumulation.

### 2.5 Gingival index

The Silness and Löe GI was used to assess the severity of gingival inflammation. As a result of clinical evaluation of gingival appearance and probing of the gingival sulcus on four gingival surfaces of all teeth (mesial, distal, labial, lingual), bleeding status was evaluated and a value between 0 and 3 will be given for each surface. Evaluations were made with the force applied with the WHO periodontal probe not exceeding 20 g and by moving it in the gingival groove. According to this evaluation index, the health of the mucosa around the teeth is scored. The classification of the GI was recognized as the absence of inflammation, mild, moderate, or severe inflammation.

### 2.6 The DII

Twenty‐four‐hour dietary records were taken for 1 day. These records were analyzed with the BEBIS program to determine the daily energy and 25 nutrients intake of the participant, including protein, carbohydrate, fiber, total fat, saturated fat, polyunsaturated fatty acids, monounsaturated fatty acids, omega‐3 fatty acids, omega‐6 fatty acids, cholesterol, vitamin A, β‐Carotene, vitamin D, vitamin E, vitamin C, thiamin, riboflavin, niacin, vitamin B12, vitamin B6, folic acid, Fe, Mg, Se, and Zn. To calculate the inflammatory load gained from daily diet, DII score was used (Shivappa et al., 2014).

## 3 RESULTS

The study group consisted of 138 participants in total (47 female [36.2%] and 83 male [63.8%]). Mean age was 32.8 ± 7.9 years.

DII values based on the percentiles for each of the 25 food parameters are shown in Table 1. The scores range from 0.73 to −2.60.

**TABLE 1:** Quartiles of the dietary inflammatory index (DII).

DII	*n* (%)
First quartile	34 (24.6)
Second quartile	35 (25.4)
Third quartile	35 (25.4)
Fourth quartile	34 (24.6)

Oral and dental examination of the participants is given in Table 2. 65.8% of the participants had absence or small amount plaque accumulation, while 39.2% had moderate or dense amount of plaque accumulation according to the PI classification. According to the GI, majority of the participants had absence or mild inflammation (86.8%), whereas moderate or severe inflammation was found in 13.2% (Table 2).

**TABLE 2:** Oral and dental examination.

Oral and dental examination	*n* (%)
PI	
Absence or small	75 (65.8)
Moderate or dense	34 (39.2)
GI	
Absence or mild	99 (86.8)
Moderate or severe	15 (13.2)

	Median	Minimum	Maximum
DMFT	4.00	0.00	20.00
PI	0.96	0.05	2.42
GI	0.70	0.06	2.10

Abbreviations: DMFT, Decayed, Missing, and Filled Teeth; GI, gingival index; PI, plaque index.

DMFT scores were found with median 4.0, and no statistically significant difference was observed between DII quartiles. Median of PI and GI scores were found to be 0.96 and 0.70, respectively, and were not statistically different between DII quartiles. While DII did not differ by PI classification, DII quartile between second and third found a difference according to the GI classification (Table 3). No statistically significant correlation was found between DII mean score and DMFT, PI, GI mean scores.

**TABLE 3:** Oral and dental examination by quartiles of the dietary inflammatory index (DII).

DII	First quartile	Second quartile	Third quartile	Fourth quartile	*p*‐Value
DMFT [median]	5.0	5.0	4.0	4.0	.265
PI (%)					
Absence or small	60.0	62.1	70.4	71.4	.814
Moderate or dense	40.0	37.9	29.6	28.6	
GI (%)					
Absence or mild	26.3^ab^	21.2^a^	27.3^b^	25.3^ab^	**.017**
Moderate or severe	26.7	53.3	0.0	20.0	

*Note*: *p* Values arise from ANOVA for continuous variables or chi‐square tests for categorical variables. Bold indicates statistically significant values (*p* < .05). Means having the different letters (a, b) in the same rows are significantly different.

Abbreviations: DMFT, Decayed, Missing, and Filled Teeth; GI, gingival index; PI, plaque index.

## 4 DISCUSSION

A relationship between the inflammatory properties of the diet and periodontal diseases could occur. Although the number of previous studies is small, results supporting that inflammatory diet may be associated with oral diseases have been reported (Machado et al., 2021). One study found that an anti‐inflammatory diet was associated with less missing teeth (Kotsakis et al., 2018), and another study reported that an anti‐inflammatory diet could reduce gingivitis (Woelber et al., 2019). In our study, we aimed to investigate the relationship between the DII of the diet and DMFT, GI, PI, and the main findings of our study were (i) no significant difference between DMFT and DII quartiles, (ii) DII did not differ by PI qualification, and (iii) significant difference between DII quartiles of second and third according to GI classification.

Dietary behaviors are suggested to be a risk factor for periodontal dise ases (Hujoel & Lingström, 2017). In a cross‐sectional study, an inverse relation between dietary fiber intake and periodontal disease among US adults ≥30 years old has been reported (Nielsen et al., 2016). In addition, improvement of periodontal disease markers by treatment with high‐fiber, low‐fat diet for 8 weeks has been shown (Kondo et al., 2014). Furthermore, higher intakes of vitamins B_6_, B_12_, C, and E, and folate, iron, potassium, and magnesium were significantly associated with a lower risk of periodontal disease (Watson et al., 2022). But these studies focused on specific nutrients, and the relation between inflammatory properties of the whole diet and periodontal diseases still remains unclear. Two recent studies have investigated interactions between DII and periodontal diseases (Li et al., 2021; Machado et al., 2021). Machado et al. (2021) demonstrated a relation between DII and periodontal probing depth, and Li et al. (2021) showed that consuming a pro‐inflammatory diet is associated with periodontal disease. These studies used periodontal probing depth as periodontal disease marker. In our study, we used DMFT for evaluating oral health and GI and PI for evaluating periodontal health, and in the contrary, we found no relation between DII and DMFT, PI. But we have found a significant difference between DII quartiles of second and third according to GI classification. Benamghar et al. (1982) have based GI on signs of inflammation (and in a recent study, improvement of gingival and plaque indices has been shown by consumption of cranberry functional beverage. But in the same study, no effect of cranberry functional beverage on systemic inflammation has been reported (Woźniewicz et al., 2018).

The new definition of periodontal health has been proposed as the absence of clinically detectable inflammation (Chapple et al., 2018), and some studies investigated the relation between systemic burden and periodontitis (Li et al., 2021). Considering that an anti‐inflammatory diet can improve systemic inflammation (Mukherjee et al., 2023), it can be said that dietary interventions are also important in the treatment of periodontal diseases. For instance, a diet rich in fiber, fruit, and vegetables and low in saturated fats and processed products lowers the production of pro‐inflammatory cytokines that may stimulate the periodontal immune response (Giugliano et al., 2006). In addition, ameliorating effect of bilberry consumption on some markers of gingival inflammation has been reported by Widén et al. (2015). On the other hand, a stone age diet for 4 weeks has been shown to increase plaque levels, but to decrease bleeding on probing and probing depth (Baumgartner et al., 2009).

This study has some limitations. First of all, sample size is small. Second, we used 24‐h dietary recall to determine food consumption and to calculate DII, but this method may not reflect overall diet of the participants. Finally, this study has a cross‐sectional design, and to evaluate the relation between anti‐inflammatory diet and oral ad periodontal health, randomized controlled trials are needed.

In this study, we evaluated the relation between pro‐inflammatory properties of diet and DMFT, GI, and PI. We used DII to determine pro‐inflammatory properties of diet and found no significant relation between DII and DMFT, PI but found a significant relation between second and third quartiles of DII for GI. To sum up according to the results of our and other's studies investigating the relation between DII and periodontal health, it seems that pro‐inflammatory properties of diet and periodontal health are related and so dietary strategies on the treatment of periodontal diseases should be considered.

## AUTHOR CONTRIBUTIONS

**Ziya Erokay Metin:** Conceptualization (equal); data curation (equal); formal analysis (equal); methodology (equal); resources (equal); writing – original draft (equal); writing – review and editing (equal). **M. Merve Tengilimoglu‐Metin:** Conceptualization (equal); data curation (equal); formal analysis (equal); investigation (equal); methodology (equal); validation (equal); writing – original draft (equal); writing – review and editing (equal). **Nuh Oğuz:** Data curation (equal); methodology (equal). **Mevlüde Kizil:** Methodology (equal).

## FUNDING INFORMATION

This research received no specific grant from any funding agency in the public, commercial, or not‐for‐profit sectors.

## CONFLICT OF INTEREST STATEMENT

The authors have declared no conflicts of interest.

## Data Availability

The data that support the findings of this study are available on request from the corresponding author.

## References

[fsn33641-bib-0001] Baumgartner, S. , Imfeld, T. , Schicht, O. , Rath, C. , Persson, R. E. , & Persson, G. R. (2009). The impact of the stone age diet on gingival conditions in the absence of oral hygiene. Journal of Periodontology, 80(5), 759–768. 19405829 10.1902/jop.2009.080376

[fsn33641-bib-0002] Benamghar, L. , Penaud, J. , Kaminsky, P. , Abt, F. , & Martin, J. (1982). Comparison of gingival index and sulcus bleeding index as indicators of periodontal status. Bulletin of the World Health Organization, 60(1), 147. 6979418 PMC2536024

[fsn33641-bib-0003] Chapple, I. L. C. , Mealey, B. L. , Van Dyke, T. E. , Bartold, P. M. , Dommisch, H. , Eickholz, P. , Geisinger, M. L. , Genco, R. J. , Glogauer, M. , Goldstein, M. , Griffin, T. J. , Holmstrup, P. , Johnson, G. K. , Kapila, Y. , Lang, N. P. , Meyle, J. , Murakami, S. , Plemons, J. , Romito, G. A. , … Yoshie, H. (2018). Periodontal health and gingival diseases and conditions on an intact and a reduced periodontium: Consensus report of workgroup 1 of the 2017 World Workshop on the Classification of Periodontal and Peri‐Implant Diseases and Conditions. Journal of Periodontology, 89, S74–S84. 29926944 10.1002/JPER.17-0719

[fsn33641-bib-0004] Dinarello, C. A. (2000). Proinflammatory cytokines. Chest, 118(2), 503–508. 10936147 10.1378/chest.118.2.503

[fsn33641-bib-0005] Frisbee, S. J. , Chambers, C. B. , Frisbee, J. C. , Goodwill, A. G. , & Crout, R. J. (2010). Self‐reported dental hygiene, obesity, and systemic inflammation in a pediatric rural community cohort. BMC Oral Health, 10(1), 1–8. 20849640 10.1186/1472-6831-10-21 PMC2954840

[fsn33641-bib-0006] Giugliano, D. , Ceriello, A. , & Esposito, K. (2006). The effects of diet on inflammation: Emphasis on the metabolic syndrome. Journal of the American College of Cardiology, 48(4), 677–685. 16904534 10.1016/j.jacc.2006.03.052

[fsn33641-bib-0007] Gondivkar, S. M. , Gadbail, A. R. , Gondivkar, R. S. , Sarode, S. C. , Sarode, G. S. , Patil, S. , & Awan, K. H. (2019). Nutrition and oral health. Disease‐a‐Month, 65(6), 147–154. 30293649 10.1016/j.disamonth.2018.09.009

[fsn33641-bib-0008] Hujoel, P. P. , & Lingström, P. (2017). Nutrition, dental caries and periodontal disease: A narrative review. Journal of Clinical Periodontology, 44, S79–S84. 28266117 10.1111/jcpe.12672

[fsn33641-bib-0009] Kleinman, R. E. , & Greer, F. R. (2014). Nutrition AA of PC on pediatric nutrition. American Academy of Pediatrics.

[fsn33641-bib-0010] Kondo, K. , Ishikado, A. , Morino, K. , Nishio, Y. , Ugi, S. , Kajiwara, S. , Kurihara, M. , Iwakawa, H. , Nakao, K. , Uesaki, S. , Shigeta, Y. , Imanaka, H. , Yoshizaki, T. , Sekine, O. , Makino, T. , Maegawa, H. , King, G. L. , & Kashiwagi, A. (2014). A high‐fiber, low‐fat diet improves periodontal disease markers in high‐risk subjects: A pilot study. Nutrition Research, 34(6), 491–498. 25026916 10.1016/j.nutres.2014.06.001

[fsn33641-bib-0011] Kotsakis, G. A. , Chrepa, V. , Shivappa, N. , Wirth, M. , Hébert, J. , Koyanagi, A. , & Tyrovolas, S. (2018). Diet‐borne systemic inflammation is associated with prevalent tooth loss. Clinical Nutrition, 37(4), 1306–1312. 28633943 10.1016/j.clnu.2017.06.001 PMC5723246

[fsn33641-bib-0012] Li, A. , Chen, Y. , Schuller, A. A. , van Der Sluis, L. W. M. , & Tjakkes, G. E. (2021). Dietary inflammatory potential is associated with poor periodontal health: A population‐based study. Journal of Clinical Periodontology, 48(7), 907–918. 33899265 10.1111/jcpe.13472 PMC8251843

[fsn33641-bib-0013] Machado, V. , Botelho, J. , Viana, J. , Pereira, P. , Lopes, L. B. , Proença, L. , Delgado, A. S. , & Mendes, J. J. (2021). Association between dietary inflammatory index and periodontitis: A cross‐sectional and mediation analysis. Nutrients, 13(4), 1194. 33916342 10.3390/nu13041194 PMC8066166

[fsn33641-bib-0014] Mahan, L. K. , & Raymond, J. L. (2016). Krause's food & the nutrition care process‐e‐book. Elsevier Health Sciences.

[fsn33641-bib-0015] Moynihan, P. J. (2005). The role of diet and nutrition in the etiology and prevention of oral diseases. Bulletin of the World Health Organization, 83, 694–699. 16211161 PMC2626331

[fsn33641-bib-0016] Mukherjee, M. S. , Han, C. Y. , Sukumaran, S. , Delaney, C. L. , & Miller, M. D. (2023). Effect of anti‐inflammatory diets on inflammation markers in adult human populations: A systematic review of randomized controlled trials. Nutrition Reviews, 81(1), 55–74. 10.1093/nutrit/nuac045 35831971

[fsn33641-bib-0017] Muniz, F. W. M. G. , Nogueira, S. B. , Mendes, F. L. V. , Rösing, C. K. , Moreira, M. M. S. M. , de Andrade, G. M. , & Carvalho, R. S. (2015). The impact of antioxidant agents complimentary to periodontal therapy on oxidative stress and periodontal outcomes: A systematic review. Archives of Oral Biology, 60(9), 1203–1214. 26067357 10.1016/j.archoralbio.2015.05.007

[fsn33641-bib-0018] Nibali, L. , Fedele, S. , D'aiuto, F. , & Donos, N. (2012). Interleukin‐6 in oral diseases: A review. Oral Diseases, 18(3), 236–243. 22050374 10.1111/j.1601-0825.2011.01867.x

[fsn33641-bib-0019] Nielsen, S. J. , Trak‐Fellermeier, M. A. , Joshipura, K. , & Dye, B. A. (2016). Dietary fiber intake is inversely associated with periodontal disease among US adults. The Journal of Nutrition, 146(12), 2530–2536. 27798338 10.3945/jn.116.237065 PMC5118764

[fsn33641-bib-0020] Okada, H. , & Murakami, S. (1998). Cytokine expression in periodontal health and disease. Critical Reviews in Oral Biology and Medicine, 9(3), 248–266. 9715365 10.1177/10454411980090030101

[fsn33641-bib-0021] Saito, T. , & Shimazaki, Y. (2007). Metabolic disorders related to obesity and periodontal disease. Periodontology 2000, 43(1), 254–266. 17214843 10.1111/j.1600-0757.2006.00186.x

[fsn33641-bib-0022] Sheiham, A. , & James, W. P. T. (2015). Diet and dental caries: The pivotal role of free sugars reemphasized. Journal of Dental Research, 94(10), 1341–1347. 26261186 10.1177/0022034515590377

[fsn33641-bib-0023] Shivappa, N. , Steck, S. E. , Hurley, T. G. , Hussey, J. R. , & Hébert, J. R. (2014). Designing and developing a literature‐derived, population‐based dietary inflammatory index. Public Health Nutrition, 17(8), 1689–1696. 23941862 10.1017/S1368980013002115 PMC3925198

[fsn33641-bib-0024] Song, I.‐S. , Han, K. , Ko, Y. , Park, Y.‐G. , Ryu, J.‐J. , & Park, J.‐B. (2016). Associations between the consumption of carbonated beverages and periodontal disease: The 2008–2010 Korea national health and nutrition examination survey. Medicine (Baltimore), 95(28), e4253. 27428235 10.1097/MD.0000000000004253 PMC4956829

[fsn33641-bib-0025] Stanski, R. , & Palmer, C. A. (2015). Oral health and nutrition as gatekeepers to overall health: We are all in this together. European Journal of General Dentistry, 4(3), 99–105.

[fsn33641-bib-0026] Takashiba, S. , Naruishi, K. , & Murayama, Y. (2003). Perspective of cytokine regulation for periodontal treatment: Fibroblast biology. Journal of Periodontology, 74(1), 103–110. 12593604 10.1902/jop.2003.74.1.103

[fsn33641-bib-0027] Watson, S. , Woodside, J. V. , Winning, L. , Wright, D. M. , Srinivasan, M. , & McKenna, G. (2022). Associations between self‐reported periodontal disease and nutrient intakes and nutrient‐based dietary patterns in the UK Biobank. Journal of Clinical Periodontology, 49(5), 428–438. 35170067 10.1111/jcpe.13604 PMC9315140

[fsn33641-bib-0028] Widén, C. , Coleman, M. , Critén, S. , Karlgren‐Andersson, P. , Renvert, S. , & Persson, G. R. (2015). Consumption of bilberries controls gingival inflammation. International Journal of Molecular Sciences, 16(5), 10665–10673. 25970751 10.3390/ijms160510665 PMC4463668

[fsn33641-bib-0029] Woelber, J. P. , Gärtner, M. , Breuninger, L. , Anderson, A. , König, D. , Hellwig, E. , al‐Ahmad, A. , Vach, K. , Dötsch, A. , Ratka‐Krüger, P. , & Tennert, C. (2019). The influence of an anti‐inflammatory diet on gingivitis. A randomized controlled trial. Journal of Clinical Periodontology, 46(4), 481–490. 30941800 10.1111/jcpe.13094

[fsn33641-bib-0030] Woźniewicz, M. , Nowaczyk, P. M. , Kurhańska‐Flisykowska, A. , Wyganowska‐Świątkowska, M. , Lasik‐Kurdyś, M. , Walkowiak, J. , & Bajerska, J. (2018). Consumption of cranberry functional beverage reduces gingival index and plaque index in patients with gingivitis. Nutrition Research, 58, 36–45. 30340813 10.1016/j.nutres.2018.06.011

